# Supplementary figures and images for: Expanding understanding of chick embryo’s nervous system development at HH22-HH41 embryonic stages using X-ray microcomputed tomography
Source: PLoS One. 2024 Nov 15;19(11):e0310426. doi: 10.1371/journal.pone.0310426 (PMC11567531; doi:10.1371/journal.pone.0310426)

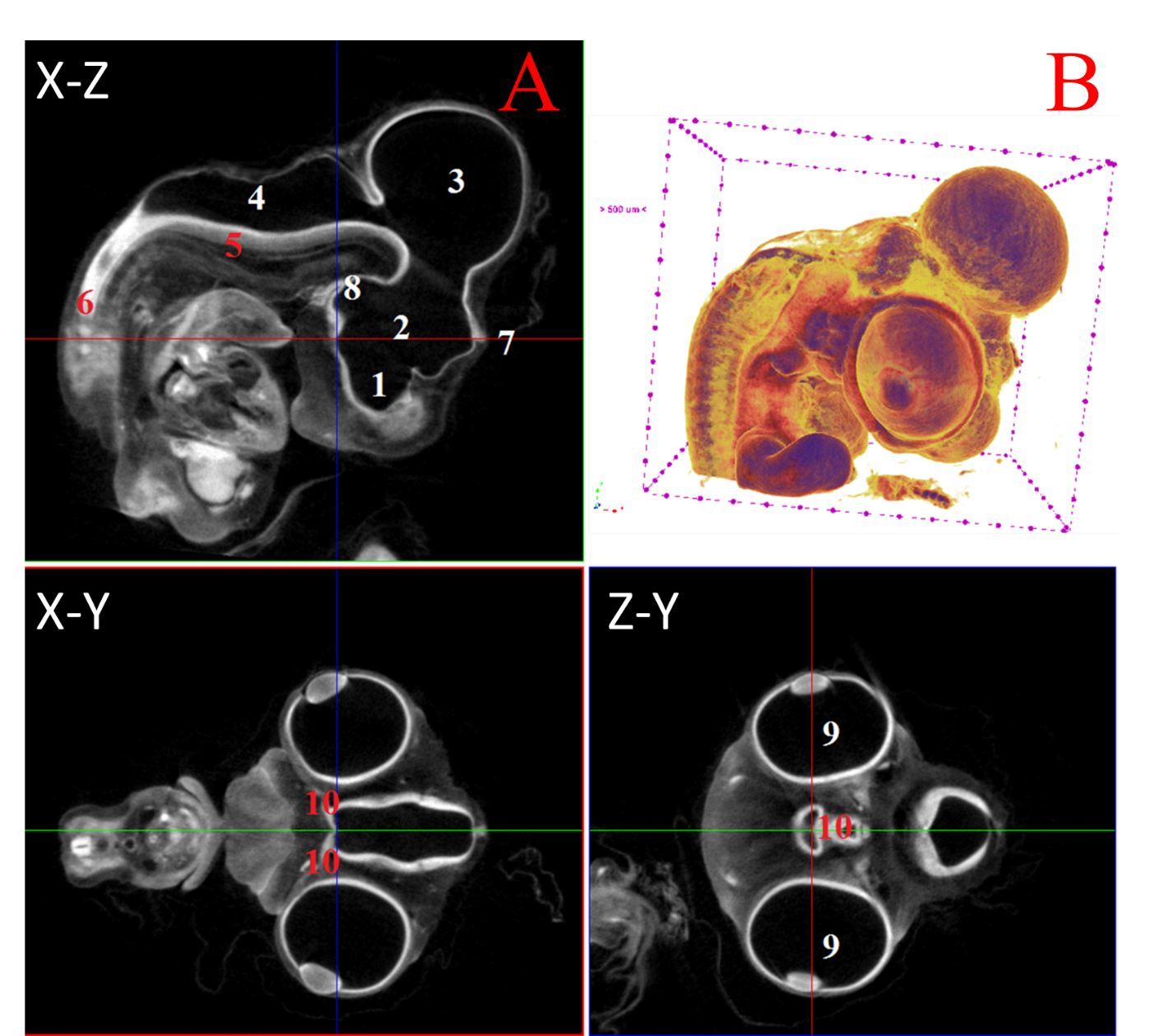

Supplement: S1 Fig — Representative cross-sectional images of the head region of a chick embryo (day 5, embryonic stages HH25-27), counterstained with 1% PTA for 24 h at 40°C (A): coronal (X-Z), transaxial (X-Y) and sagittal (Z-Y) planes and isosurface 3D renderings of the head region (B). The following structures are marked on the images: 1 –telencephalon (lat. telencephalon); 2 –diencephalon (lat. diencephalon); 3 –midbrain (lat. mesencephalon); 4 –hindbrain (lat. rhombencephalon); 5 –medulla oblongata (lat. myelencephalon); 6 –spinal cord (lat. medulla spinalis); 7 –pineal gland (lat. corpus pineale); 8 –pituitary gland (lat. hypophysis); 9 –eyes (lat. oculus); 10 –chiasma and optic nerves (lat. chiasma opticum). Visualization of structures in the DataViewer software and CTvox software. (TIF) [file pone.0310426.s001.tif]

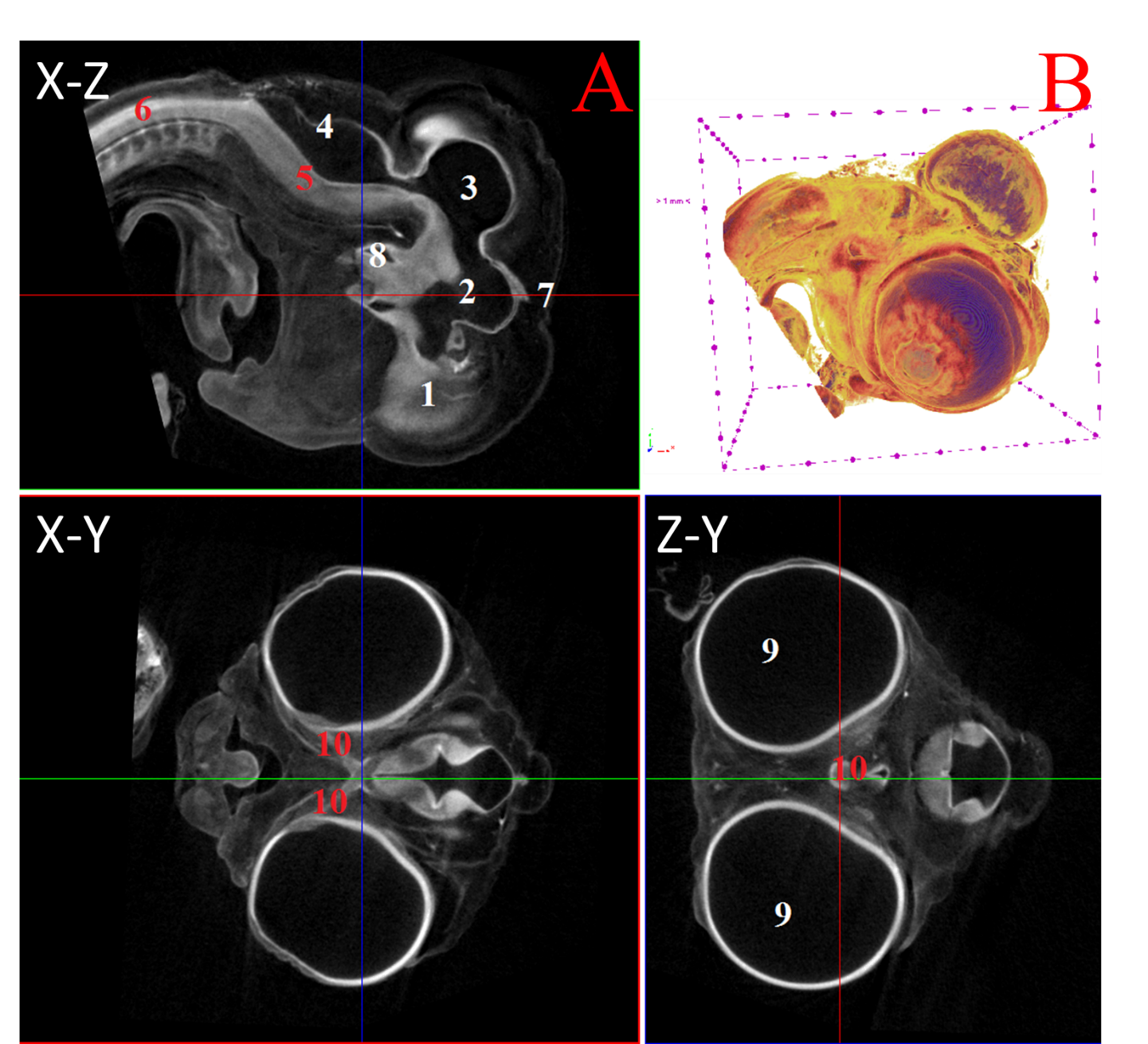

Supplement: S2 Fig — Representative cross-sectional images of the head region of a chick embryo (day 7, embryonic stages HH30-32), counterstained with 1% PTA for 24 h at 40°C (A): coronal (X-Z), transaxial (X-Y) and sagittal (Z-Y) planes and isosurface 3D renderings of the head region (B). The following structures are marked on the images: 1 –telencephalon (lat. telencephalon); 2 –diencephalon (lat. diencephalon); 3 –midbrain (lat. mesencephalon); 4 –hindbrain (lat. rhombencephalon); 5 –medulla oblongata (lat. myelencephalon); 6 –spinal cord (lat. medulla spinalis); 7 –pineal gland (lat. corpus pineale); 8 –pituitary gland (lat. hypophysis); 9 –eyes (lat. oculus); 10 –chiasma and optic nerves (lat. chiasma opticum). Visualization of structures in the DataViewer software and CTvox software. (TIF) [file pone.0310426.s002.tif]

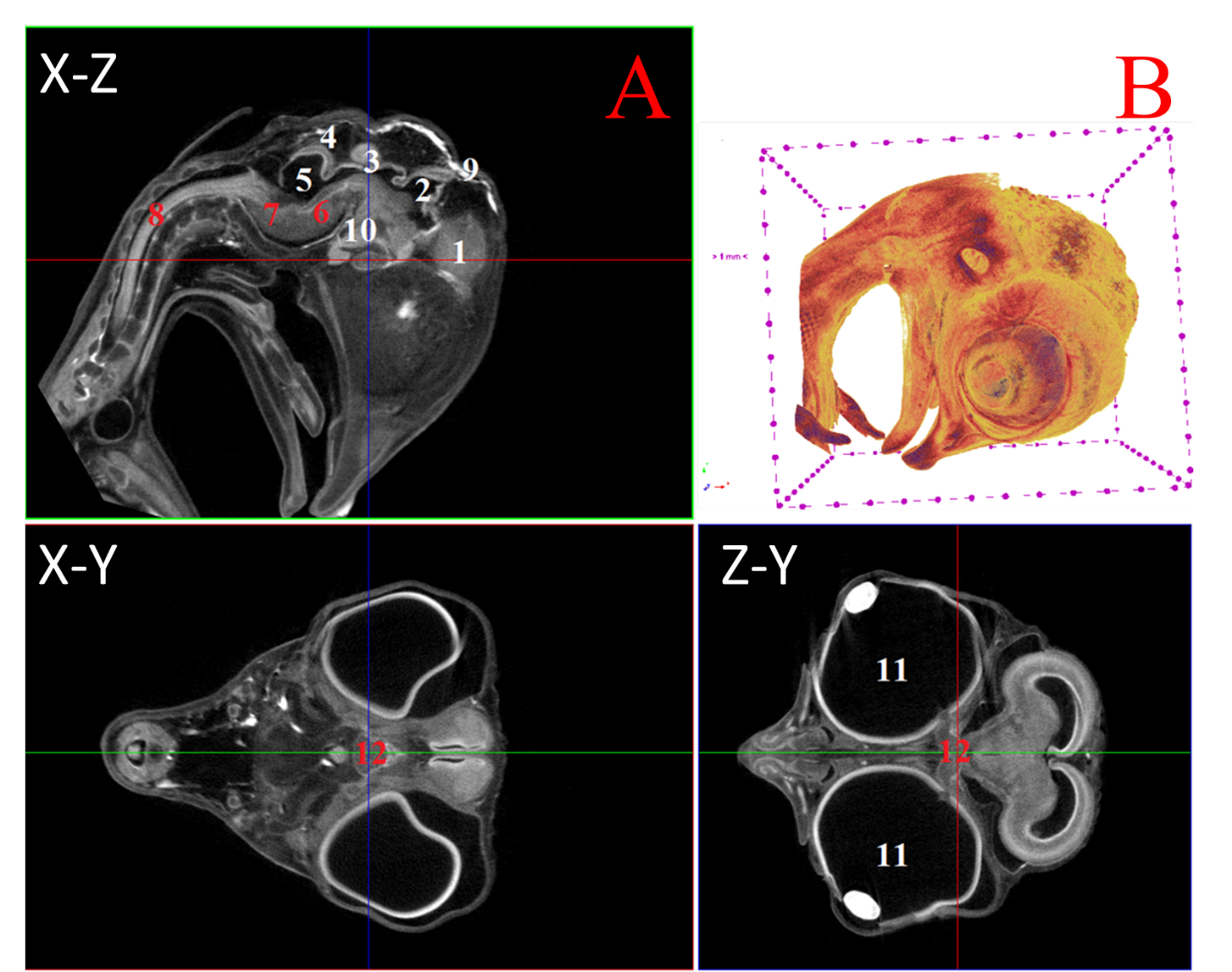

Supplement: S3 Fig — Representative cross-sectional images of the head region of a chick embryo (day 9, embryonic stage HH35), counterstained with 1% PTA for 96 h at 40°C (A): coronal (X-Z), transaxial (X-Y) and sagittal (Z-Y) planes and isosurface 3D renderings of the head region (B). The following structures are marked on the images: 1 –telencephalon (lat. telencephalon); 2 –diencephalon (lat. diencephalon); 3 –midbrain (lat. mesencephalon); 4 –сerebellum (lat. cerebellum); 5 –fourth ventricle (lat. ventriculus quartus); 6 –pons (lat. pons); 7 –medulla oblongata (lat. myelencephalon); 8 –spinal cord (lat. medulla spinalis); 9 –pineal gland (lat. corpus pineale); 10 –pituitary gland (lat. hypophysis); 11 –eyes (lat. oculus); 12 –chiasma and optic nerves (lat. chiasma opticum). Visualization of structures in the DataViewer software and CTvox software. (TIF) [file pone.0310426.s003.tif]

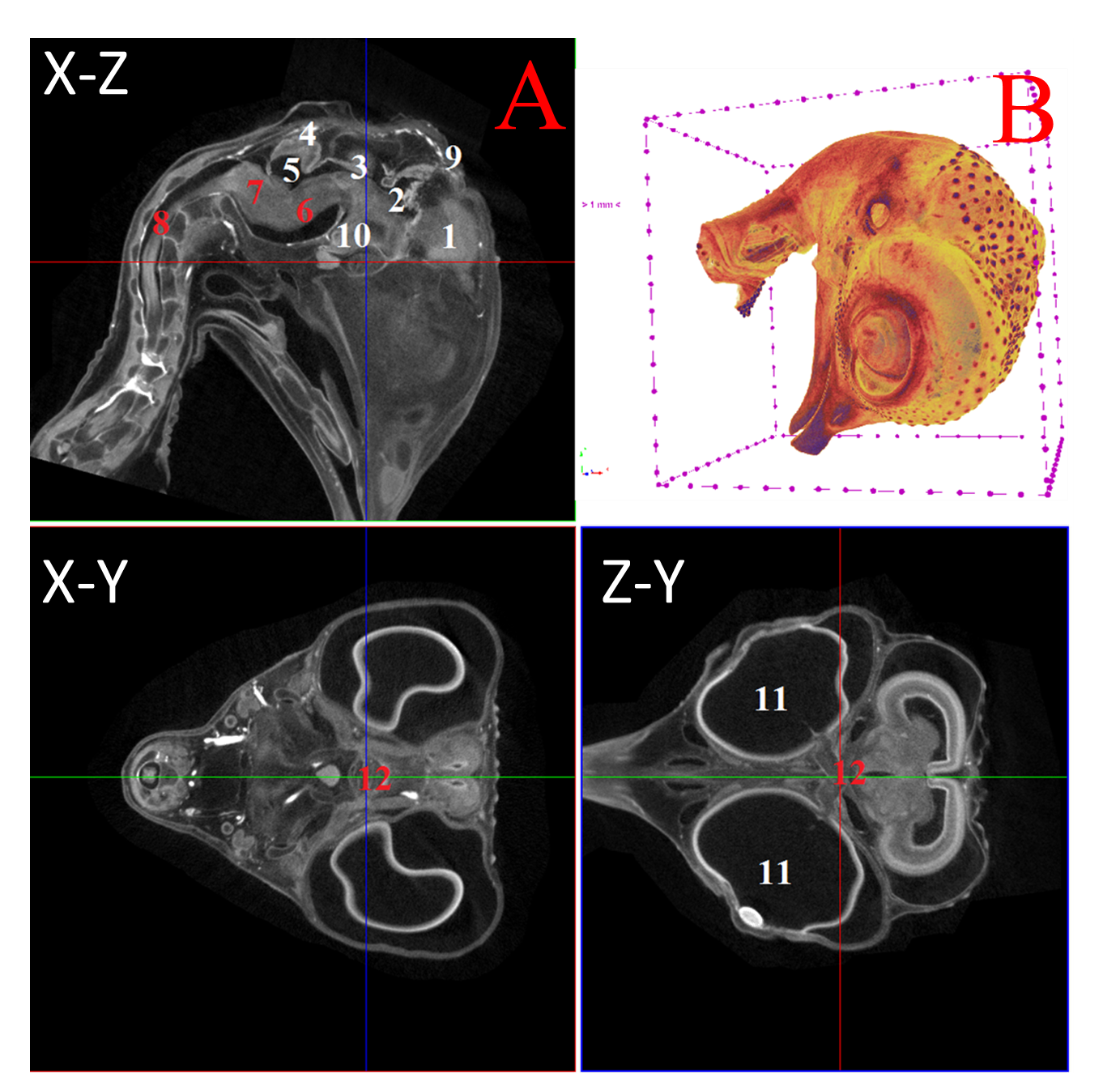

Supplement: S4 Fig — Representative cross-sectional images of the head region of a chick embryo (day 10, embryonic stage HH36), counterstained with 1% PTA for 96 h at 40°C (A): coronal (X-Z), transaxial (X-Y) and sagittal (Z-Y) planes and isosurface 3D renderings of the head region (B). The following structures are marked on the images: 1 –telencephalon (lat. telencephalon); 2 –diencephalon (lat. diencephalon); 3 –midbrain (lat. mesencephalon); 4 –сerebellum (lat. cerebellum); 5 –fourth ventricle (lat. ventriculus quartus); 6 –pons (lat. pons); 7 –medulla oblongata (lat. myelencephalon); 8 –spinal cord (lat. medulla spinalis); 9 –pineal gland (lat. corpus pineale); 10 –pituitary gland (lat. hypophysis); 11 –eyes (lat. oculus); 12 –chiasma and optic nerves (lat. chiasma opticum). Visualization of structures in the DataViewer software and CTvox software. (PNG) [file pone.0310426.s004.png]

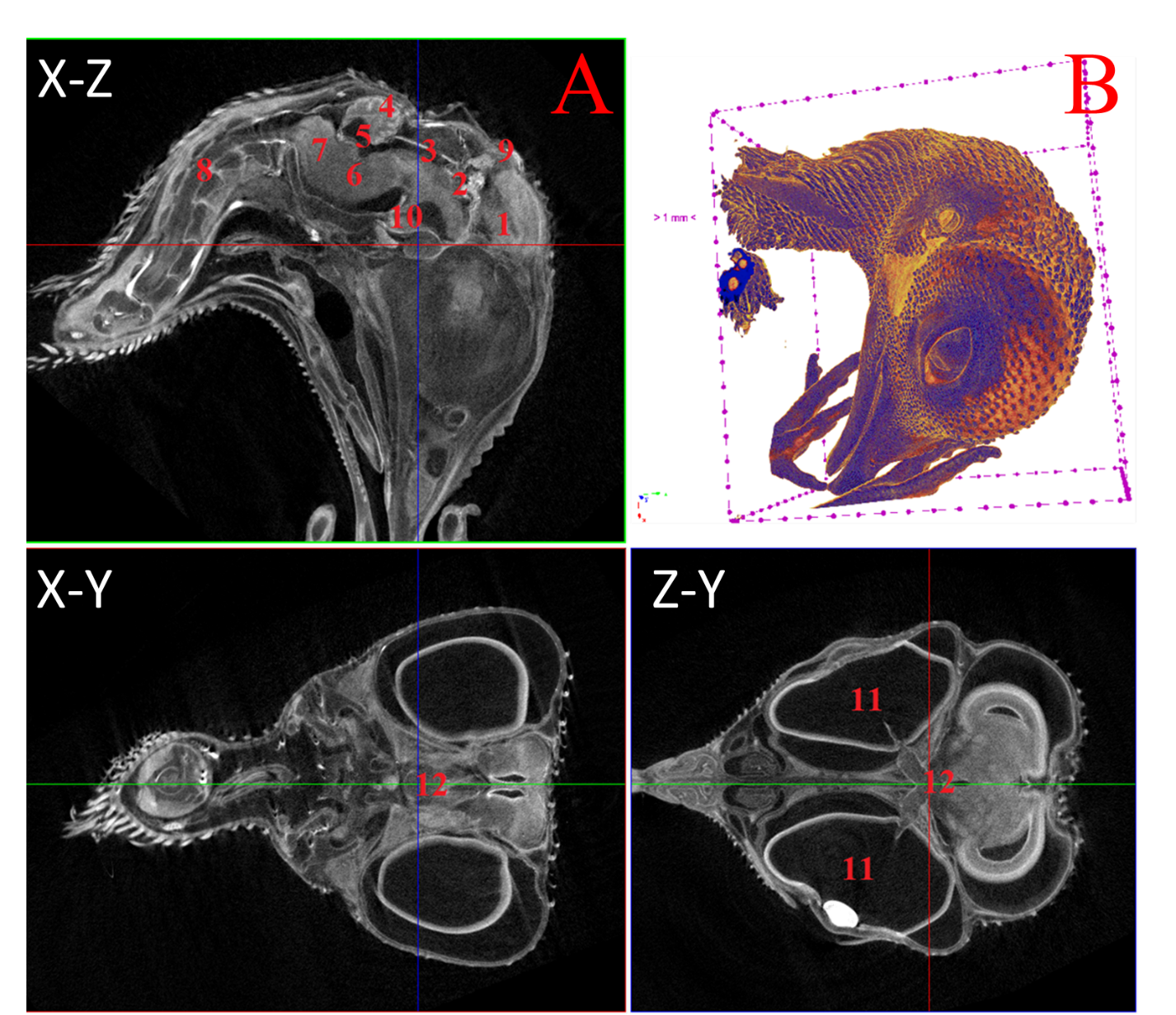

Supplement: S5 Fig — Representative cross-sectional images of the head region of a chick embryo (day 12, embryonic stage HH38), counterstained with 1% PTA for 96 h at 40°C (A): coronal (X-Z), transaxial (X-Y) and sagittal (Z-Y) planes and isosurface 3D renderings of the head region (B). The following structures are marked on the images: 1 –telencephalon (lat. telencephalon); 2 –diencephalon (lat. diencephalon); 3 –midbrain (lat. mesencephalon); 4 –сerebellum (lat. cerebellum); 5 –fourth ventricle (lat. ventriculus quartus); 6 –pons (lat. pons); 7 –medulla oblongata (lat. myelencephalon); 8 –spinal cord (lat. medulla spinalis); 9 –pineal gland (lat. corpus pineale); 10 –pituitary gland (lat. hypophysis); 11 –eyes (lat. oculus); 12 –chiasma and optic nerves (lat. chiasma opticum). Visualization of structures in the DataViewer software and CTvox software. (PNG) [file pone.0310426.s005.png]

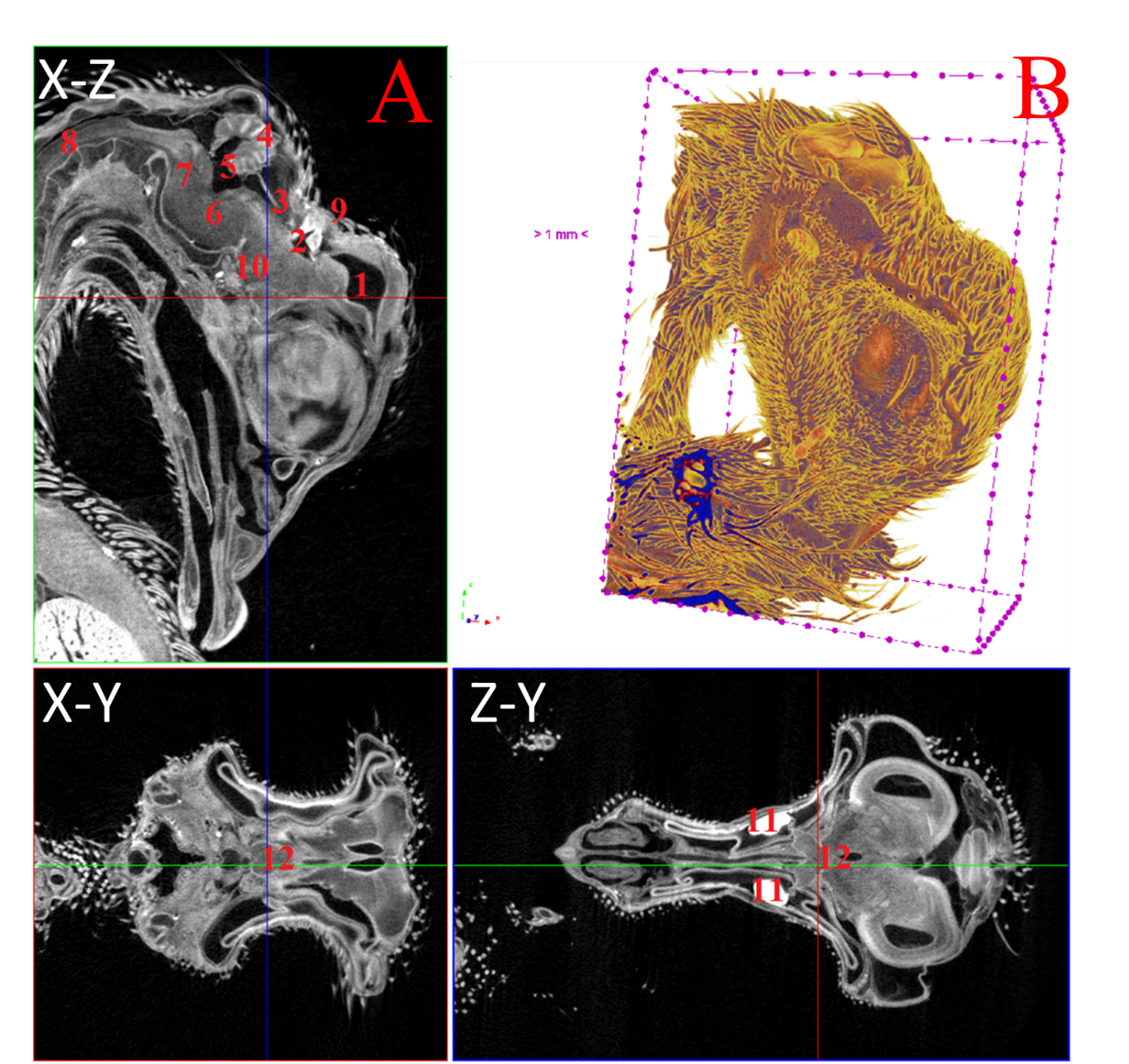

Supplement: S6 Fig — Representative cross-sectional images of the head region of a chick embryo (day 13, embryonic stage HH39), counterstained with 1% PTA for 96 h at 40°C (A): coronal (X-Z), transaxial (X-Y) and sagittal (Z-Y) planes and isosurface 3D renderings of the head region (B). The following structures are marked on the images: 1 –telencephalon (lat. telencephalon); 2 –diencephalon (lat. diencephalon); 3 –midbrain (lat. mesencephalon); 4 –сerebellum (lat. cerebellum); 5 –fourth ventricle (lat. ventriculus quartus); 6 –pons (lat. pons); 7 –medulla oblongata (lat. myelencephalon); 8 –spinal cord (lat. medulla spinalis); 9 –pineal gland (lat. corpus pineale); 10 –pituitary gland (lat. hypophysis); 11 –eyes (lat. oculus); 12 –chiasma and optic nerves (lat. chiasma opticum). Visualization of structures in the DataViewer software and CTvox software. (PNG) [file pone.0310426.s006.png]

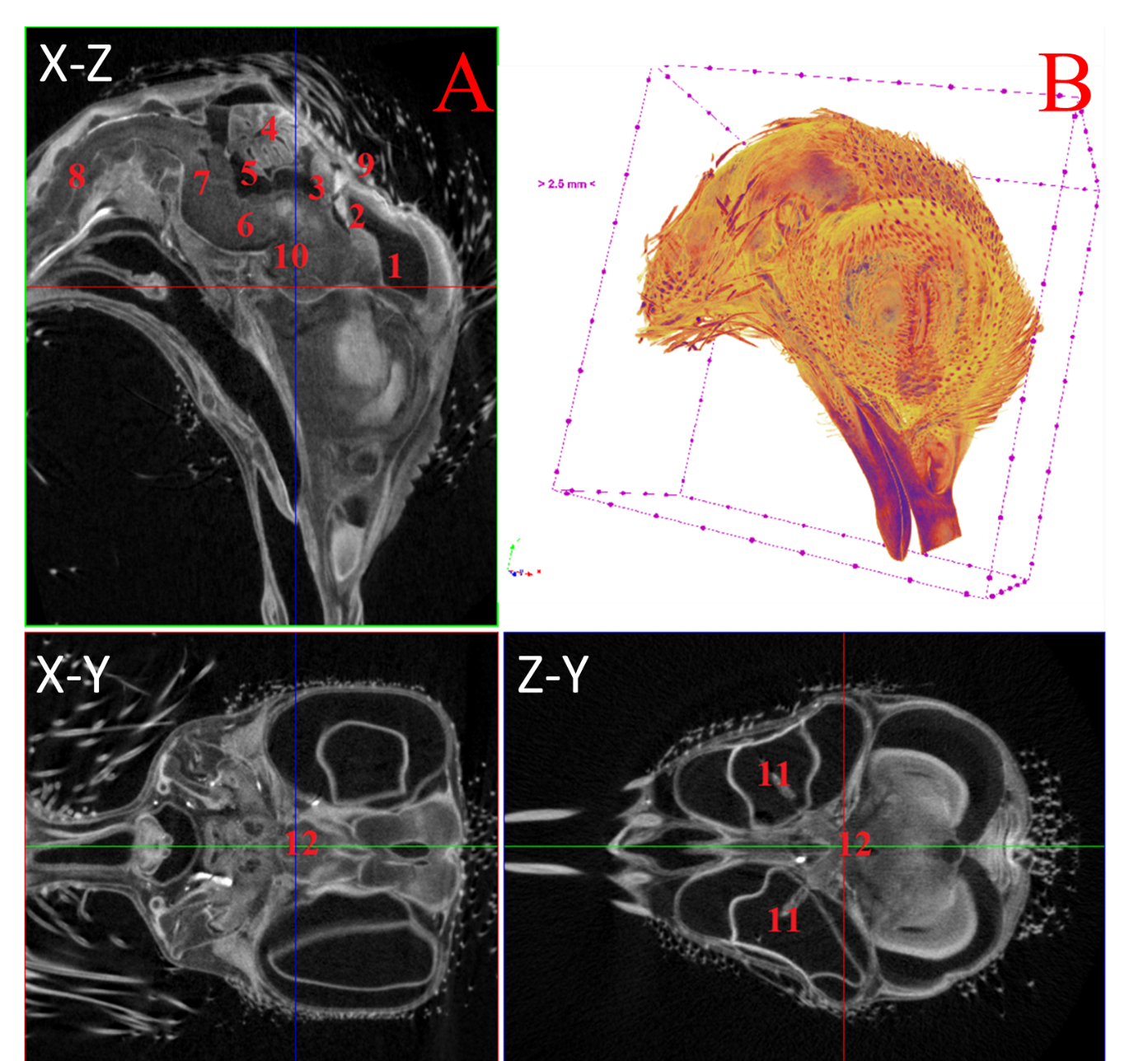

Supplement: S7 Fig — Representative cross-sectional images of the head region of a chick embryo (day 15, embryonic stage HH41), counterstained with 1% PTA for 96 h at 40°C (A): coronal (X-Z), transaxial (X-Y) and sagittal (Z-Y) planes and isosurface 3D renderings of the head region (B). The following structures are marked on the images: 1 –telencephalon (lat. telencephalon); 2 –diencephalon (lat. diencephalon); 3 –midbrain (lat. mesencephalon); 4 –сerebellum (lat. cerebellum); 5 –fourth ventricle (lat. ventriculus quartus); 6 –pons (lat. pons); 7 –medulla oblongata (lat. myelencephalon); 8 –spinal cord (lat. medulla spinalis); 9 –pineal gland (lat. corpus pineale); 10 –pituitary gland (lat. hypophysis); 11 –eyes (lat. oculus); 12 –chiasma and optic nerves (lat. chiasma opticum). Visualization of structures in the DataViewer software and CTvox software. (PNG) [file pone.0310426.s007.png]

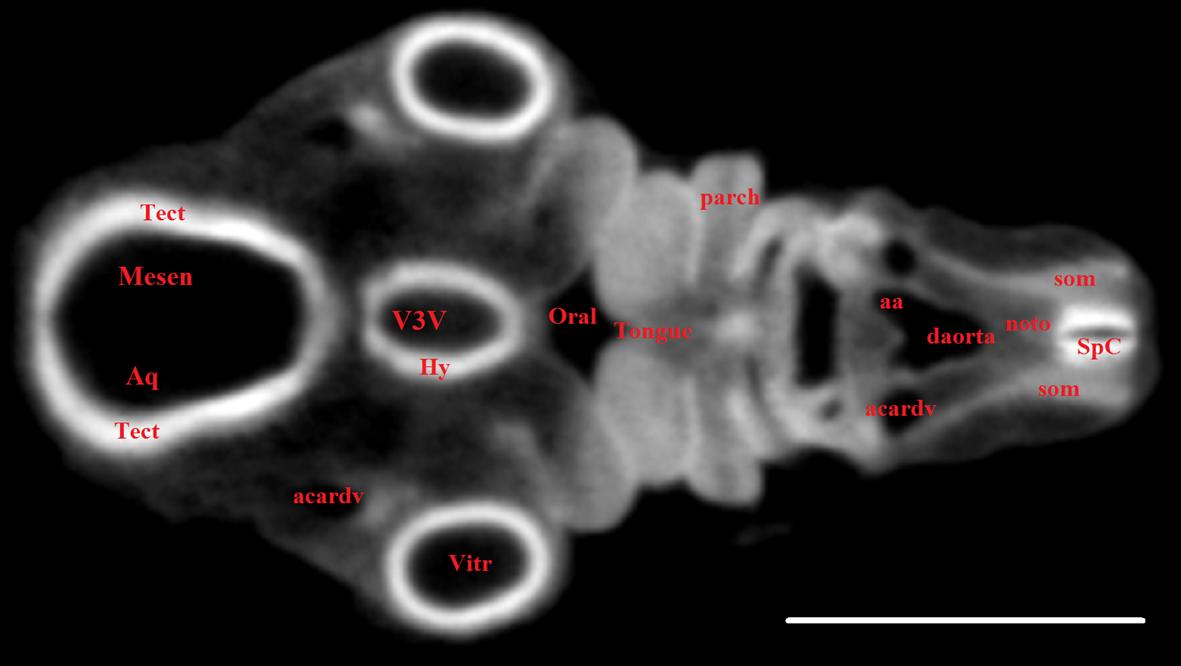

Supplement: S8 Fig — Scale ruler—1 mm. The following structures are marked on the images: AA–aortic arch artery; Acardv–anterior cardinal vein; Aq–cerebral aqueduct; Daorta–dorsal aorta; Hy–hypothalamus Mesen–mesencephalon; Noto–notochord; Oral–oral cavity; Parch–pharyngeal arch; Som–somite; SpC–spinal cord; Tect–tectum; Tongue–tongue; V3V –ventral third ventricle; Vitr–vitreous humor of eye. (PNG) [file pone.0310426.s008.png]

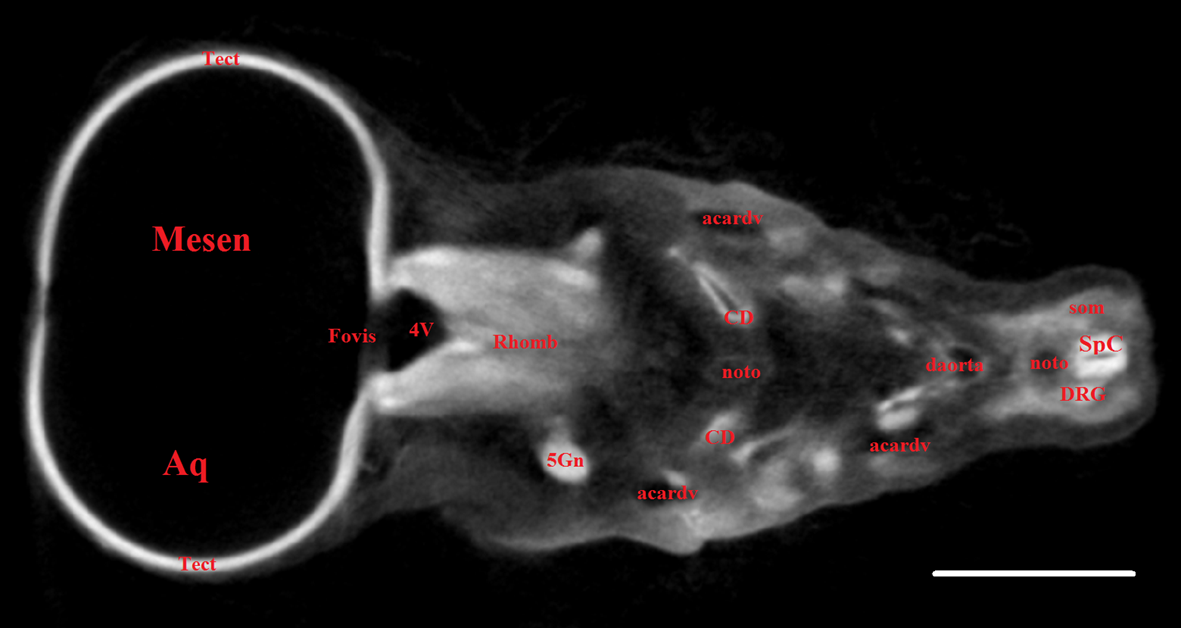

Supplement: S9 Fig — Scale ruler—1 mm. The following structures are marked on the images: 4V –fourth ventricle; 5Gn–trigeminal ganglion; Acardv–anterior cardinal vein; Aq–cerebral aqueduct; CD–cochlear duct; Daorta–dorsal aorta; DRG–dorsal root ganglion; Fovis–fovea of isthmus; Mesen–mesencephalon; Noto–notochord; Rhomb–rhombencephalon; Som–somite; SpC–spinal cord; Tect–tectum. (PNG) [file pone.0310426.s009.png]

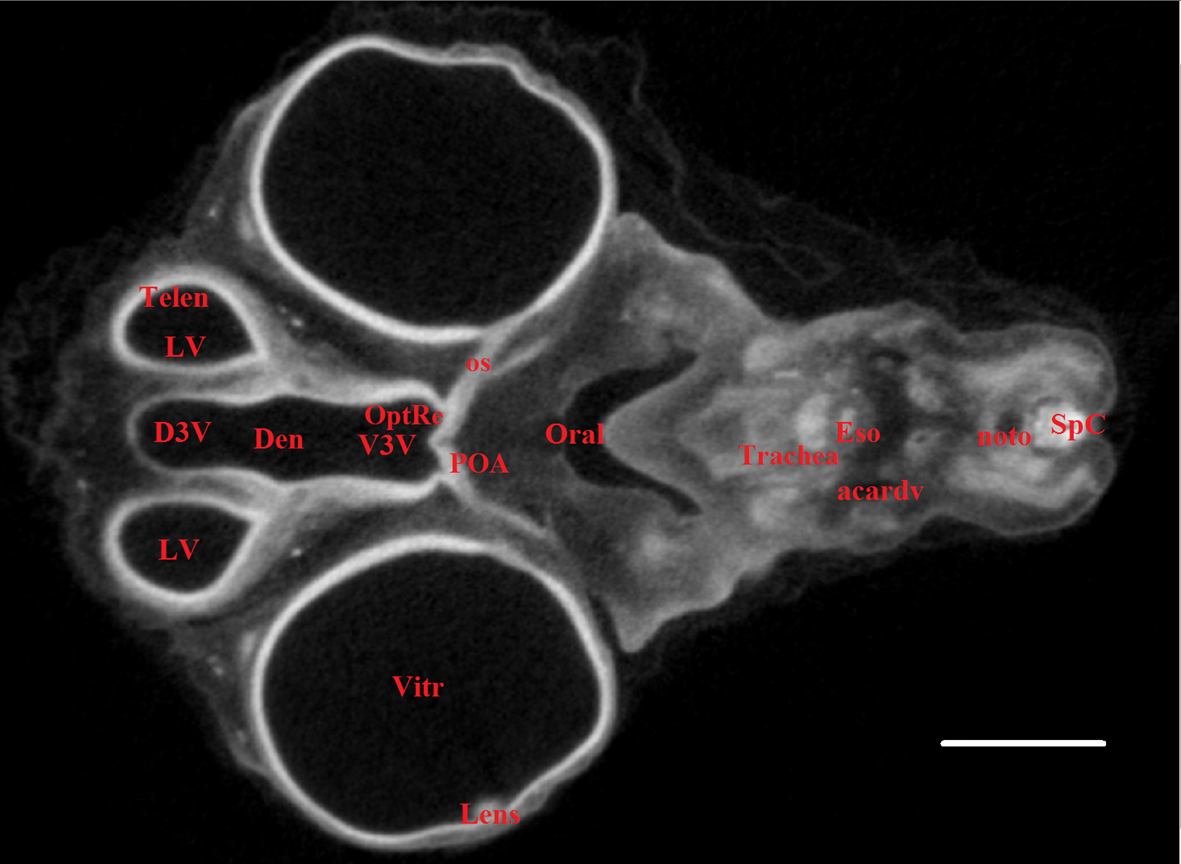

Supplement: S10 Fig — Scale ruler—1 mm. The following structures are marked on the images: Acardv–anterior cardinal vein; D3V –dorsal third ventricle; Dien–diencephalon; Eso–esophagus; Lens–lens; LV–lateral ventricle; Noto–notochord; OptRe–optic recess of 3rd ventricle. Oral–oral cavity; Os–optic stalk; SpC–spinal cord; Telen–telencephalon; Tongue–tongue; Trachea–trachea; V3V –ventral third ventricle; Vitr–vitreous humor of eye. (PNG) [file pone.0310426.s010.png]

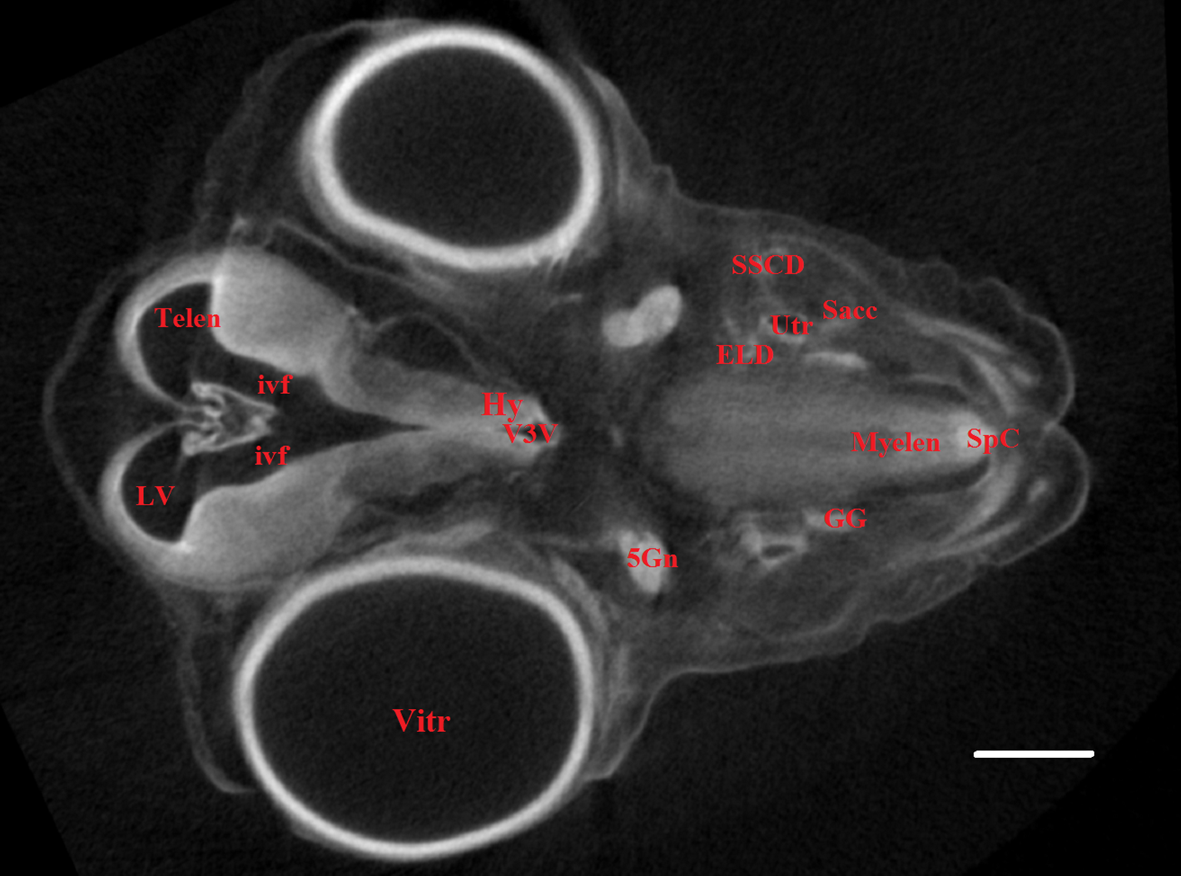

Supplement: S11 Fig — Scale ruler—1 mm. The following structures are marked on the images: 5Gn–trigeminal ganglion; ELD–endolymphatic duct; GG–glossopharyngeal ganglion; Hy–hypothalamus; Ivf–interventricular foramen of Monro; LV–lateral ventricle; Myelen–myelencephalon; Sacc–saccule of inner ear; SpC–spinal cord; SSCD–superior semicircular duct; Telen–telencephalon; Utr–utricle of inner ear; V3V –ventral third ventricle; Vitr–vitreous humor of eye. (PNG) [file pone.0310426.s011.png]

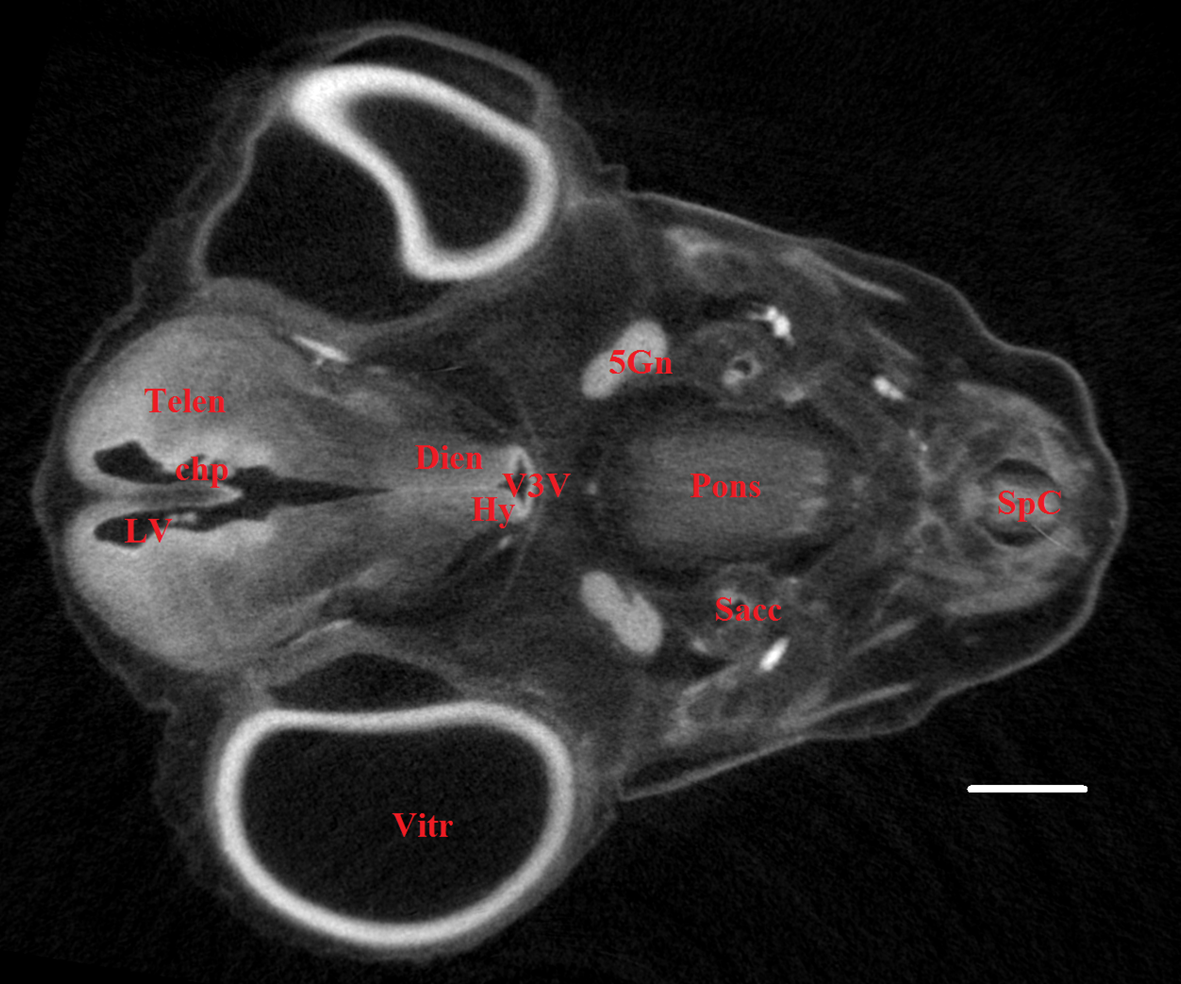

Supplement: S12 Fig — Scale ruler—1 mm. The following structures are marked on the images: 5Gn–trigeminal ganglion; Chp–choroid plexus; Dien–diencephalon; Hy–hypothalamus; LV–lateral ventricle; Pons–pons; Sacc–saccule of inner ear; SpC–spinal cord; V3V –ventral third ventricle; Vitr–vitreous humor of eye. (PNG) [file pone.0310426.s012.png]

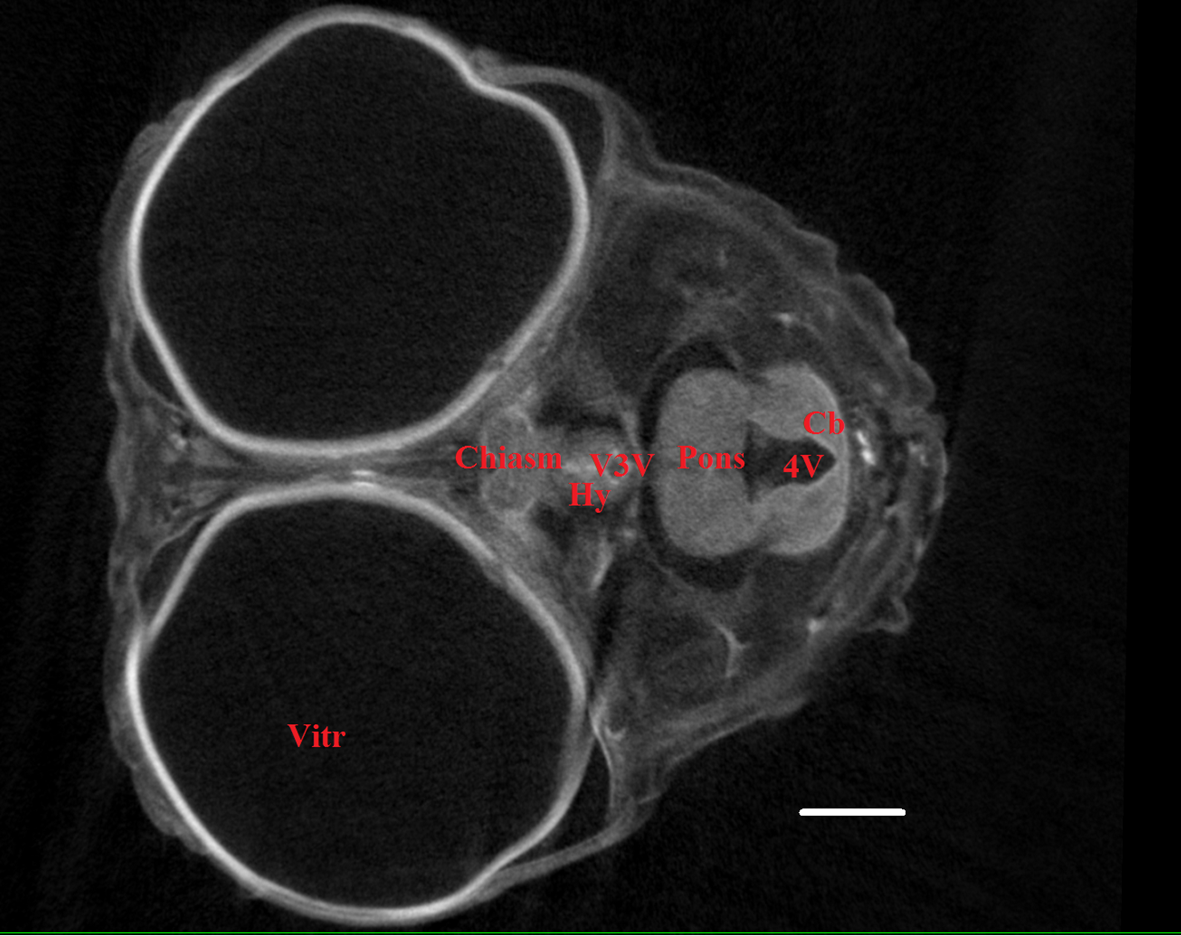

Supplement: S13 Fig — Scale ruler—1 mm. The following structures are marked on the images: 4V –fourth ventricle; Cb–сerebellum; Chiasma–optic chiasma; Hy–hypothalamus; Pons–pons; V3V –ventral third ventricle; Vitr–vitreous humor of eye. (PNG) [file pone.0310426.s013.png]

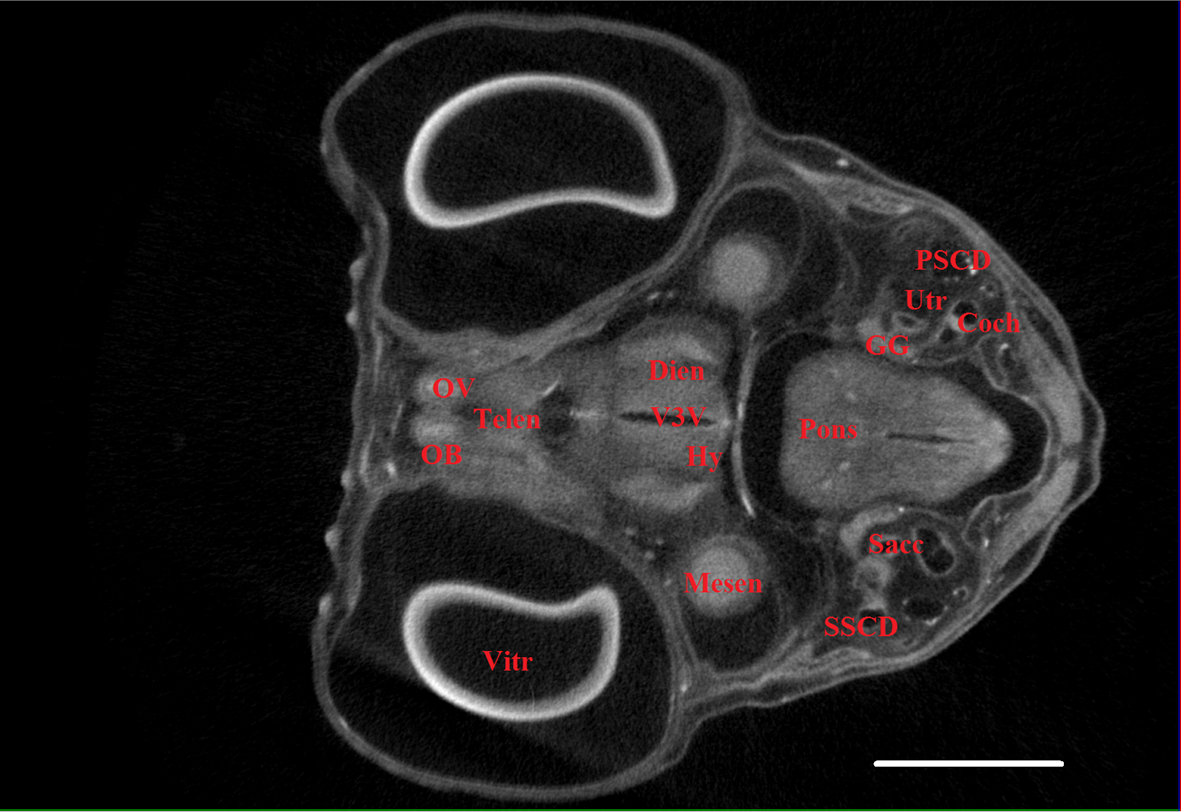

Supplement: S14 Fig — Scale ruler—2 mm. The following structures are marked on the images: Coch–cochlea; Dien–diencephalon; GG–glossopharyngeal ganglion; Hy–hypothalamus; Mesen–mesencephalon; OB–olfactory bulb; OV–olfactory ventricle; Pons–pons; PSCD–posterior semicircular duct; Sacc–saccule of inner ear; SSCD–superior semicircular duct; Telen–telencephalon; Utr–utricle of inner ear; V3V –ventral third ventricle; Vitr–vitreous humor of eye. (TIF) [file pone.0310426.s014.tif]

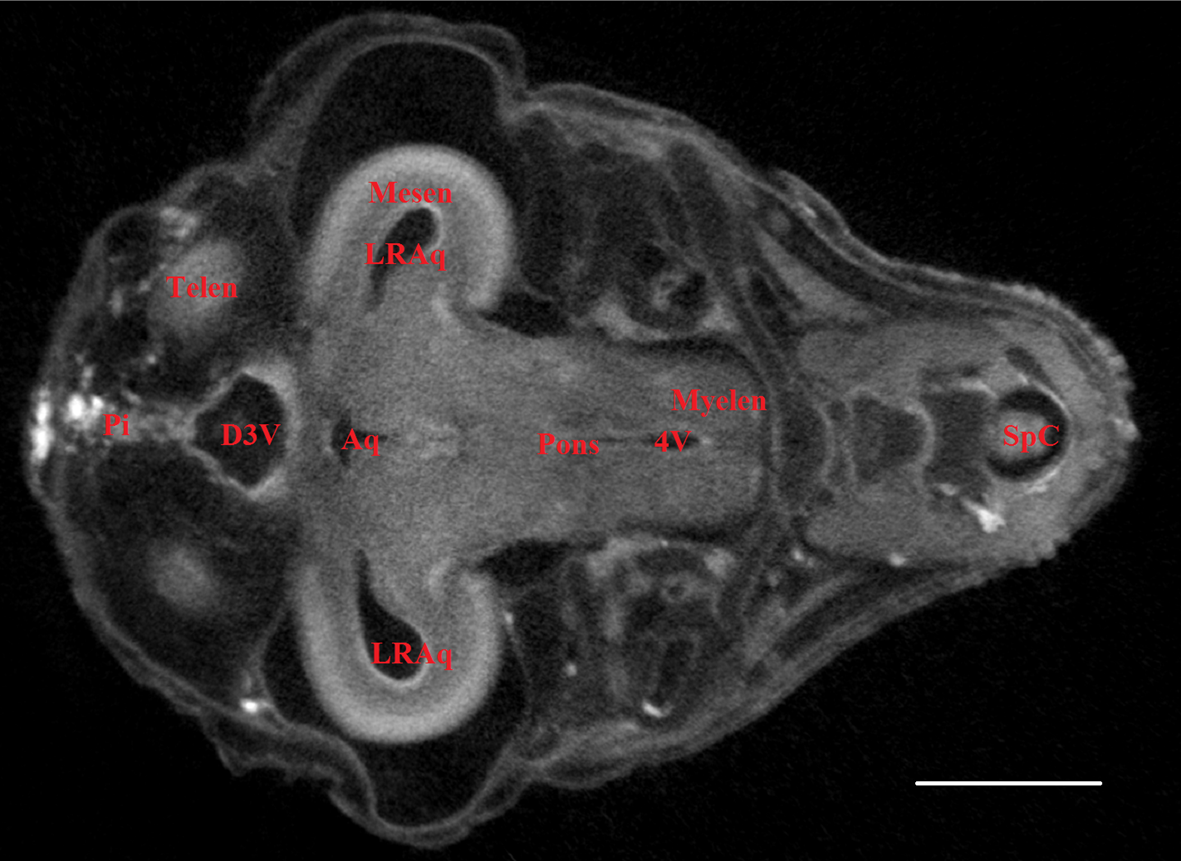

Supplement: S15 Fig — Scale ruler—2 mm. The following structures are marked on the images: 4V –fourth ventricle; Aq–cerebral aqueduct; D3V –dorsal third ventricle; LRAq–lateral recess of the cerebral aqueduct; Mesen–mesencephalon; Myelen–myelencephalon; Pi–pineal gland; Pons–pons; SpC–spinal cord; Telen–telencephalon. (TIF) [file pone.0310426.s015.tif]

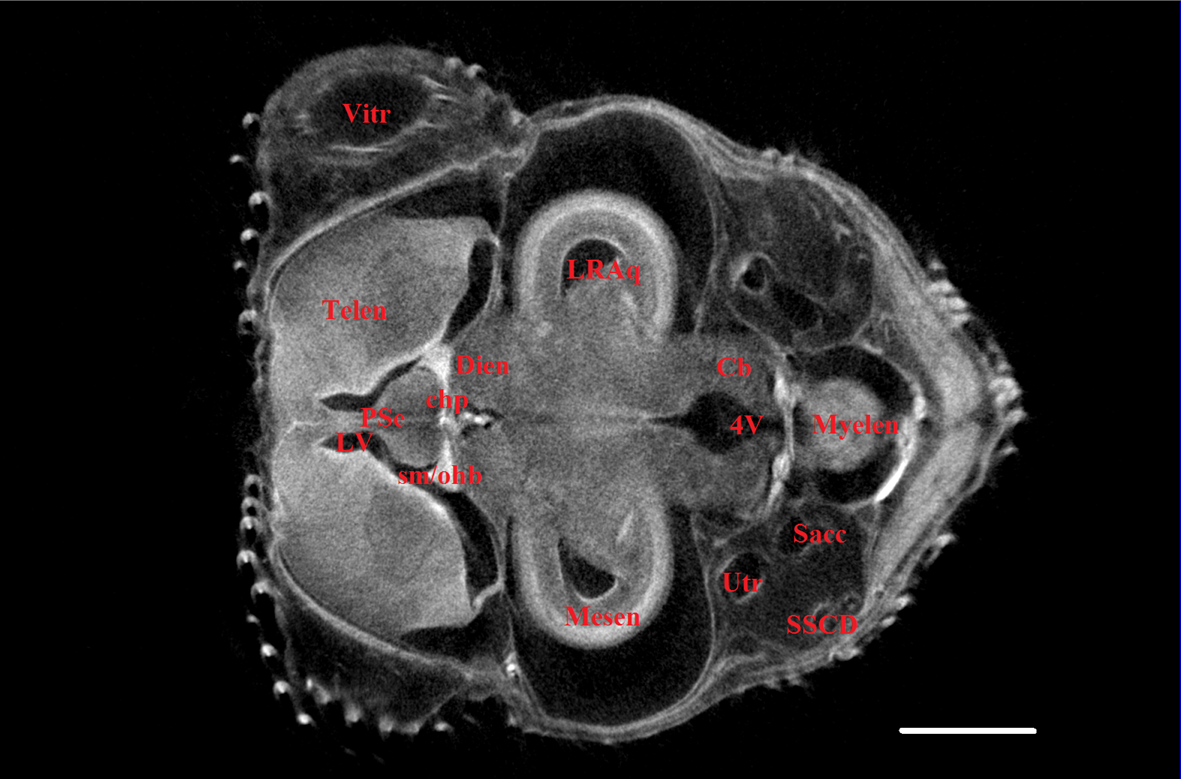

Supplement: S16 Fig — Scale ruler—2 mm. The following structures are marked on the images: 4V –fourth ventricle; Cb–сerebellum; Chp–choroid plexus; Dien–diencephalon; LRAq–lateral recess of the cerebral aqueduct; LV–lateral ventricle; Mesen–mesencephalon; Myelen–myelencephalon; PSe–pallial septum; Sacc–saccule of inner ear; Sm/ohb–stria medullaris talami/olfactohabenular tract; SSCD–superior semicircular duct; Telen–telencephalon; Utr–utricle of inner ear; Vitr–vitreous humor of eye. (TIF) [file pone.0310426.s016.tif]

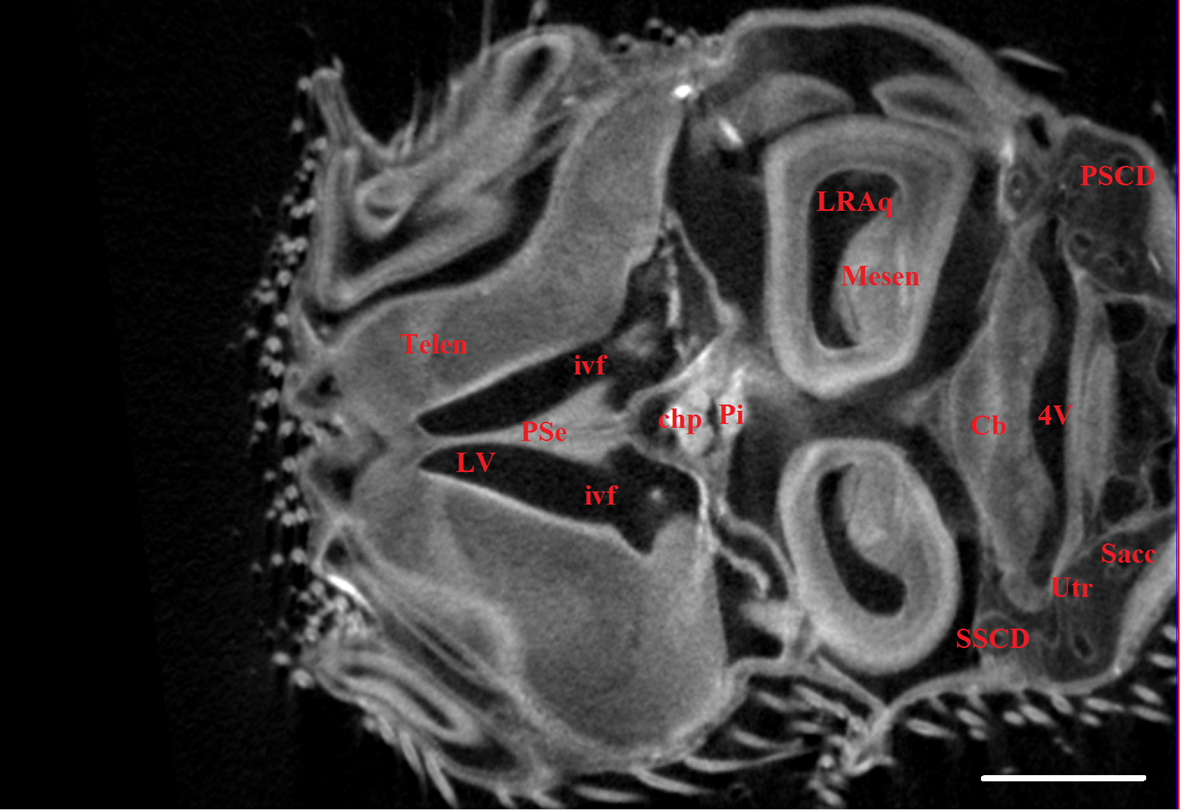

Supplement: S17 Fig — Scale ruler—2 mm. The following structures are marked on the images: 4V –fourth ventricle; Cb–сerebellum; Chp–choroid plexus; Ivf–interventricular foramen of Monro; LRAq–lateral recess of the cerebral aqueduct; LV–lateral ventricle; Mesen–mesencephalon; Pi–pineal gland; PSCD–posterior semicircular duct; PSe–pallial septum; Sacc–saccule of inner ear; SSCD–superior semicircular duct; Telen–telencephalon; Utr–utricle of inner ear. (PNG) [file pone.0310426.s017.png]

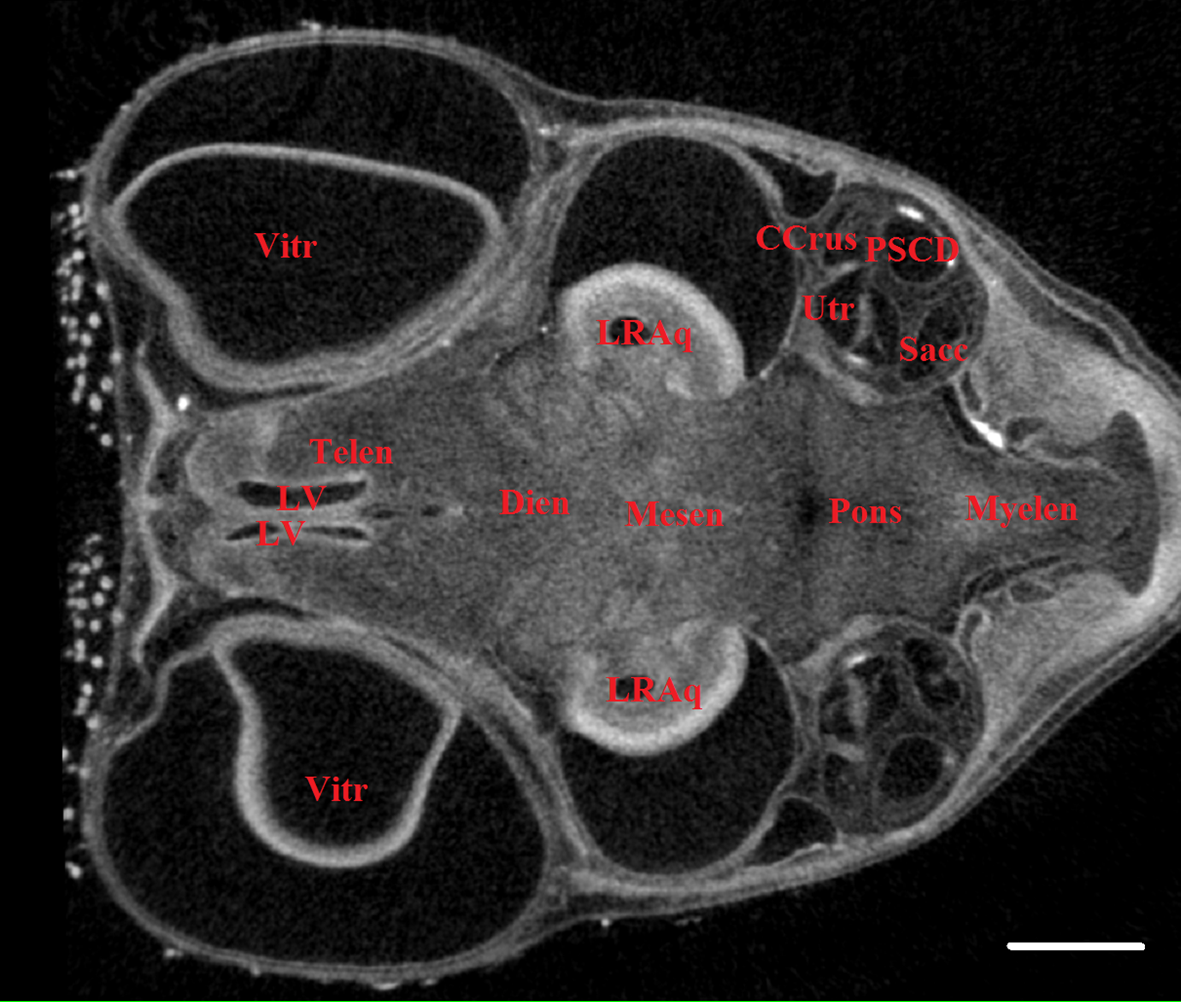

Supplement: S18 Fig — Scale ruler—2 mm. The following structures are marked on the images: CCrus–common crus of semicircular duct; Dien–diencephalon; LRAq–lateral recess of the cerebral aqueduct; LV–lateral ventricle; Mesen–mesencephalon; Myelen–myelencephalon; Pons–pons; PSCD–posterior semicircular duct; Sacc–saccule of inner ear; Telen–telencephalon; Utr–utricle of inner ear; Vitr–vitreous humor of eye. (PNG) [file pone.0310426.s018.png]

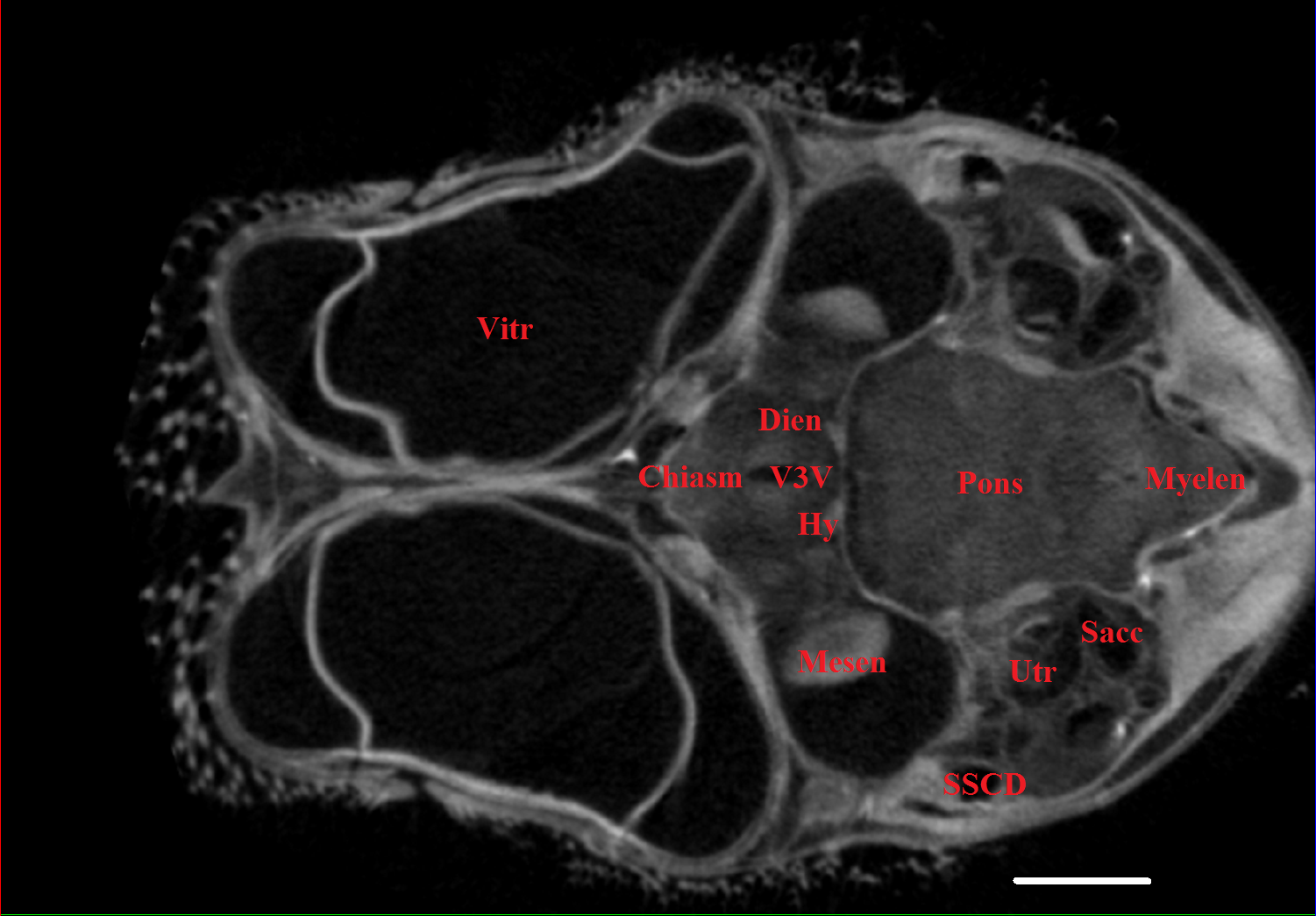

Supplement: S19 Fig — Scale ruler—2 mm. The following structures are marked on the images: Chiasma–optic chiasma; Dien–diencephalon; Hy–hypothalamus; Mesen–mesencephalon; Myelen–myelencephalon; Pons–pons; Sacc–saccule of inner ear; SSCD–superior semicircular duct; Utr–utricle of inner ear; V3V –ventral third ventricle; Vitr–vitreous humor of eye. (PNG) [file pone.0310426.s019.png]
